# Busulfan Chemotherapy Downregulates TAF7/TNF-α Signaling in Male Germ Cell Dysfunction

**DOI:** 10.3390/biomedicines12102220

**Published:** 2024-09-28

**Authors:** Daoyuan Huang, Zhenbo Tu, Antoine E. Karnoub, Wenyi Wei, Abdol-Hossein Rezaeian

**Affiliations:** Department of Pathology, Beth Israel Deaconess Medical Center, Harvard Medical School, Boston, MA 02215, USA

**Keywords:** Busulfan, chemotherapy, TAF7, TNF-α, stem cell, spermatogonia, infertility, inflammation

## Abstract

**Background**: Busulfan is an FDA-approved alkylating drug used in the chemotherapy of advanced acute myeloid leukemia. The precise mechanisms by which Busulfan kills spermatogonia stem cells (SSCs) are not yet completely understood. **Methods**: Using a murine model, we evaluated Busulfan-induced apoptosis and DNA damage signaling between testis and ovary tissues. We executed RT-qPCR, analyzed single-nuclei RNA sequencing data and performed in situ hybridization for the localization of the gene expression in the tissues. **Results**: The results indicate that, in contrast to female germ cells, haploid male germ cells undergo significant apoptosis following Busulfan chemotherapy. Moreover, a gene enrichment analysis revealed that reactive oxygen species may activate the inflammatory response in part through the TNF-α/NF-κB signaling pathway. Interestingly, in the testis, the mRNA levels of *TNF-α* and *TAF7* (TATA box-binding protein-associated factor 7) are downregulated, and testosterone levels suppressed. Mechanistically, the promoter of *TNF-α* has a conserved motif for binding TAF7, which is necessary for its transcriptional activation and may require further in-depth study. We next analyzed the tumorigenic function of *TAF7* and revealed that it is highly overexpressed in several types of human cancers, particularly testicular germ cell tumors, and associated with poor patient survival. Therefore, we executed in situ hybridization and single-nuclei RNA sequencing, finding that less *TAF7* mRNA is present in SSCs after chemotherapy. **Conclusions:** Thus, our data indicate a possible function of TAF7 in the regulation of SSCs and spermatogenesis following downregulation by Busulfan. These findings may account for the therapeutic effects of Busulfan and underlie its potential impact on cancer chemotherapy prognosis.

## 1. Introduction

Busulfan (Myleran^®^) is an alkylating antineoplastic agent that has been approved by the U.S. Food and Drug Administration (FDA) for treating advanced acute myeloid leukemia (CML) [1]. Although Busulfan was displaced by the new gold standard, imatinib, for the chemotherapeutic treatment of CML [2,3], it remains in use due to its relatively low cost. Busulfan has also recently been used to investigate the function of platelet-transported serotonin in liver regeneration [4]. However, it increases toxicity in the treatment of hepatic veno-occlusive disease (VOD) and sinusoidal obstruction syndrome (SOS) [5,6,7]. Given the increased use of high-dose chemotherapy following autologous or allogeneic stem cell transplantation to treat leukemias, the fertility prospects of young patients are also improving [1]. Although a large fraction of patients who receive Busulfan chemotherapy become infertile [8], the mechanism for this remains unclear and may be associated with numerous factors. For example, hormones from the pituitary gland, gonads, and uterus preserve cell survival and inhibit apoptosis to maintain fertility [9,10,11], which might be affected by Busulfan treatment.

Busulfan has variable impacts on male fertility in humans. In one study, 21 out of 26 patients treated with Busulfan after bone marrow transplantation had detectable sperm; however, 10 patients experienced oligospermia [12]. Despite this, six out of seven male patients attempting to conceive were successful, and 12% of men experienced low testosterone levels [12]. In contrast, in male patients, low-dose Busulfan after allogeneic hematopoietic stem cell transplantation had minimal impact on male reproductive function, with only 6% showing reduced testosterone levels. Of 13 male patients who attempted to be fathers, 92% were successful, with 20 children born without congenital defects [13]. Moreover, experimental data in animal models further support these findings. In rats, low-dose Busulfan effectively induces azoospermia [14]. Similarly, in mice, fertility was lost within four weeks post-treatment, with over 95% of spermatogonia stem cells (SSCs) eliminated within three days. However, the numbers of SSCs gradually recovered over time and returned to 30% of pre-treatment levels, and sperm counts reached 20%, irrespective of the Busulfan dose. Together, these findings suggest that Busulfan-induced fertility loss and recovery are closely tied to the depletion and regeneration of SSCs. Thus, understanding the threshold of SSC recovery could be key in developing clinical strategies for the preservation of male fertility after chemotherapy.

Tumor necrosis factor (TNF) is a pro-inflammatory cytokine that plays a multifunctional and critical role in several cellular events such as cell survival, apoptosis, proliferation, and differentiation [15,16]. Thus, TNF-α might induce either apoptosis or survival in a cell type-specific or cell context-dependent manner. On the one hand, TNF activates NF-kappaB (NF-κB), a heterodimeric transcription factor that can translocate into the nucleus and regulate the transcription of several proteins involved in cell survival, proliferation, and inflammation [17,18,19]. On the other hand, TNF-α induces apoptosis through binding with TNF-receptor (TNFR) 1 or TNFR2 [20,21]. Biologically, the activation of TNFR1 on immune cells induces apoptosis in several cell types, including macrophages, B cells, and T cells [21,22,23,24]. The signaling of cell death is relatively weak when induced by TNF compared to other family members of the TNF superfamily ligands such as FasL [25], in which apoptosis signaling is influenced by the antiapoptotic effects of NF-κB [26]. The stimulation of cytokines [27], the levels of reactive oxygen species (ROS) [28], and activation of caspases that can cleave several components of the NF-κB signaling pathway (RIP, IKK, etc.) [29], can shift the balance in support of either the apoptosis signaling or the survival pathway in the tissue microenvironment. For example, TNF is expressed and secreted by inflammatory cells, which may lead to inflammation-associated carcinogenesis [15]. Furthermore, TNF-α contributes to impaired IL-12 synthesis, partly through TNFR2, to enhance TGF-β (transforming growth factor β) secretion in antigen-presenting cells (APCs) [30]. Part of the homoeostatic function of TGF-β is the cell type-specific induction of apoptosis, which occurs in several cell types including B cells [31,32]. TGF-β and TNF-α are also shown to have synergistic and antagonistic effects on tumor regulation [33].

Lastly, transcription initiation factor TFIID subunit 7 is a protein encoded by the *TAF7* gene [34]. It is a component of the TFIID protein complex that binds to the TATA box, via its subunit TBP, in type II promoters to recruit RNA polymerase II and several other transcription activators in assembling the pre-initiation complex (PIC) [35]. Hence, TAF7 forms a promoter DNA-binding subcomplex of TFIID, along with TAF1 and TAF2 [35]. Although the exact function of TAF7 is currently unknown, multiple studies have revealed an association with the c-Jun signaling pathway [36]. Moreover, TAF7 may regulate human diseases such as chronic laryngitis and azoospermia [37]. Indeed, polymorphism in the *TAF7* gene increases the risk of azoospermia and male infertility in the Bengali population [37]. Moreover, evidence shows that TAF7 plays multiple antiapoptotic roles. For example, TGF-β induces the degradation of the TFIID subunit TAF7 in mouse mammary epithelial cells. This degradation is mediated by the ubiquitin ligase TRIM26, which ubiquitinates TAF7, targeting it for proteasomal degradation. TGF-β-induced cell cycle arrest and apoptosis are at least partly driven by this TRIM26-mediated TAF7 degradation [38]. Additionally, silencing *TAF7* in clear cell renal cell carcinoma (ccRCC) cells inhibits their proliferation, reduces migration, and promotes apoptosis, further supporting the role of TAF7 in cell survival mechanisms [39]. However, the connection between *TAF7* expression in spermatogonia stem cells and antiapoptotic function remains unclear.

Here, we investigate whether Busulfan chemotherapy has a unique mechanism of action on male versus female fertility in mice. We analyze the expression of several biomarkers and signaling pathways, such as those for DNA damage and TNF-α/NF-kB, in the testis compared to the ovary in females. We assess, in part, the mechanism of action of Busulfan in the regulation of testis-specific cell types for male infertility. Finally, we analyze *TAF7* and *TNF* deficiencies in spermatogonia stem cells, which may induce apoptosis in male germ cells in response to decreased testosterone levels in the blood.

## 2. Materials and Methods

### 2.1. Mice Samples and Tissue Preparation

Eighty-eight male and female ICR mice (weight 30–40 g) were used in total. Eight-week-old mice received a single intraperitoneal injection of Busulfan (40 mg/kg body weight) diluted in 1:1 DMSO–water. Tissues derived from Busulfan-injected and DMSO-injected (control) mice, including testis and ovary tissues, were excised at the end of each week for weeks 8–15 of treatment. These tissues were used for RNA extraction and/or providing formalin-fixed paraffin-embedded tissue block for H&E staining, in situ hybridization (ISH), and TUNEL assays. The mice received humane care as outlined in the Guidelines (National Institute of Agrobiological Sciences Care Committee, Tsukuba, Japan).

### 2.2. TdT-Mediated dUTP-X Nicked-End Labeling (TUNEL) Assay

The paraffin-embedded tissue sections were rehydrated (after deparaffinization) and washed in distilled water for 10 min. The sections were incubated for 15 min with proteinase K (20 μg/mL) at room temperature and washed twice with PBS (5 min). Endogenous peroxidase activity was blocked using 2% H_2_O_2_ for 5 min. Sections were rewashed 3 times with PBS (for 5 min each) and incubated for 60 min at 37 °C in a moist chamber with TUNEL mix (0.3 U/μL calf thymus terminal deoxynucleotidyl transferase, 7 pmol/μl biotin dUTP, and 1 mM cobalt chloride in 1X reaction buffer in distilled water). After washing, the sections were saturated in 2% BSA for 10 min at room temperature and then treated for 30 min at 37 °C in a moist chamber with a 1:2000 dilution of streptavidin-POD conjugate (Roche, IN, USA). After washing, the detection was performed with DAB (1.24 mg of DAB, 25 μL of 3% NiCl_2_, and 152 μL of 1 M Tris-HCl (pH 7.5) in 2 mL of distilled water) and slides were mounted in crystal mount (Biomeda, Foster City, CA, USA).

### 2.3. Quantitative Real-Time PCR

Total RNA was extracted from the prepared testis and ovary tissues using the RNeasy Mini Kit (Qiagen, Tokyo, Japan) [40,41]. An aliquot of each RNA sample was mixed with the quantity of the RNA fragment synthesized from the pEGFP-C1 vector (Invitrogen, Tokyo, Japan) needed to attain a final amount of 5 × 10^−5^ pmol/10 μg total RNA, and the resulting mixture was subjected to cDNA synthesis using a random hexamer primer (Takara BIO, Shiga, Japan) and Avian Myeloblastosis Virus-Reverse Transcriptase (Promega, Madison, WI). Primer pairs were designed using the Primer3 program based on cDNA sequences registered in GenBank (Supplementary Table S1), and the PCR products were sequenced using the respective primer pairs. Finally, an aliquot of each cDNA sample was mixed with a Real-Time PCR Master Mix solution containing SYBR Green, as recommended by the manufacturer (QPK-212, TOYOBO, Osaka, Japan), and 20 μM of the respective genes and *EGFP* primer pairs. The resulting mixtures were subjected to PCR cycles of 1 min at 95 °C followed by 40 cycles each for 15 sec at 95 °C and 60 °C for 60 sec. Data were generated using the Applied Biosystems 7500 Fast real-time PCR System (Applied Biosystems, Foster City, CA, USA) and normalized using values of *EGFP*.

### 2.4. In Situ Hybridization

Designing digoxigenin (DIG)-labeled cRNA probes and the procedure of in situ hybridization were performed as previously described [40,41]. A 122 nt probe was chosen from cDNA sequences of mouse *TAF7* transcript obtained from GenBank using G-probe software version 14.0.1 (Genetyx Co. Tokyo, Japan) (Supplementary Table S2). Briefly, the hybridization was performed in a solution containing 50% formamide, 2xSSC, 1.0 mg/mL tRNA, 1.0 mg/mL salmon sperm DNA,1.0 mg/mL BSA, 1.0% SDS, and 3.0 μg/mL DIG-labeled RNA probe at 42 °C for 26–64 h. A 120 nt lambda phage sequence (*LNE*) was used as a negative control RNA probe. In addition, the adult mouse testis produces hybridization signals when probed with DIG-labeled mouse protamine 1 antisense RNA, which serves as a positive control. Hybridization signals were then detected using the NBT/BCIP system and photographed through a Mirax microscope (Carl Zeiss, Jena, Germany).

### 2.5. Western Blot Analysis

Ovary and decapsulated testes from mice were snap-frozen in liquid nitrogen and homogenized in lysis buffer, 50 mM of Tris-HCl (pH 7.5), 4% CHAPS (3-[(3-cholamidopropyl) dimethyl-ammonio]-1-propane sulfonate), and protease inhibitor cocktail (5 μL/0.1 g, Sigma, St. Louis, MO, USA). Protein extracts were analyzed using 10% SDS-PAGE and electroblotted to PVDF membranes. Membranes were treated with antibodies for TAF7 (Proteintech, Rosemont, IL, USA, #13506-1-AP) and TNF-α (Cell Signaling, Danvers, MA, USA, #3707), followed by peroxidase-conjugated anti-rabbit secondary antibody (Abcam, Cambridge, UK; 1:2000). An anti-actin antibody (Abcam, 1:1000) was used to verify equal protein loading. Signals were visualized using an ECL kit (Amersham, Uppsala, Sweden).

### 2.6. Measurement of Total Nitric Oxide (NO)/Nitrite/Nitrate

To measure nitrites, serum was filtered using a 5000 MW cutoff filter, diluted twice, and then assayed (n = 3) at a wavelength of 540 nm, as described in the manufacturer’s instructions (R&D Systems, Minneapolis, MN, USA). The endogenous nitrite present in the serum was subtracted from the total converted nitrite to measure nitrate concentrations (in μmol/L).

### 2.7. Enzyme Immunoassay (EIA)

Serum was collected from control and Busulfan-injected male and female mice. All samples were purified through diethyl ether extraction and assayed 3 times at a wavelength of 405 nm. The EIA of estradiol and testosterone was performed according to the manufacturer’s procedure (Cayman, Ann Arbor, MI, USA).

### 2.8. GEO Data Analysis

The Gene Expression Omnibus (GEO) is a database repository of high-throughput gene expression data and hybridization arrays, chips, and microarrays. We retrieved and analyzed the gene sets corresponding to accession numbers GSE182727 and GSE86228, which are publicly available in the GEO. We executed a gene set enrichment analysis (GSEA) followed by Preranked analyses, since they selectively differentiate different treatment groups. In GSE182727, we applied a cutoff value of FC ≥ 1.5 with *p* < 0.05 for up-differentially expressed genes (DEGs), and FC ≤ 0.5 with *p* < 0.05 for down-DEGs based on Busulfan treatment compared to control treatment. In GSE86228, we applied a cutoff value of FC ≥ 2 for up-DEGs and FC ≤ 0.5 for down-DEGs based on the high and low TAF7 expression to find the corresponding heatmap for each enriched gene set.

### 2.9. Bioinformatics

The single-cell type clusters of the *TAF7* gene (ENSG00000178913) were analyzed in testis and ovary tissues using human protein atlas data (https://www.proteinatlas.org, accessed on 1 February 2024). The single-cell RNA sequencing results were visualized using a UMAP plot and represented as a bar chart. The individual cells were colored according to (1) % of max in five different bins and intervals, (2) a fixed interval of the read count, and (3) the average read count of all cells inside the hexagon. Alignment and phylogenetic reconstructions were performed using the function “build” of ETE3 3.1.2 [42] as implemented on GenomNet (https://www.genome.jp/tools/ete/, accessed on 1 March 2024). We performed multiple alignments of sequences including those from *Drosophila*, zebrafish, *Gallus gallus* (chicken), *Sus scrofa* (pig), *Canis lupus* (dog), *Bos taurus* (cow), *Ovis aries* (sheep), and *Homo sapiens* (human). A distance-based tree was inferred using the BioNJ algorithm [43] in PhyML_v20160115 [44]. The phylogram includes labels for both branch and leaf length. Branch supports are the Chi2-based parametric values returned by the approximate likelihood ratio test. The differential gene expression analysis of *TAF7* and *TNF* was performed using RNA-Seq-based data in TNMplot (https://tnmplot.com, accessed on 1 March 2024) [45]. We ran a multi-gene analysis in comparing *TAF7* and *TNF* gene expression between normal, tumor, and metastatic tissues, and the resulting data are presented as a density plot. We conducted a pan-cancer RNA-Seq analysis of *TAF7* and *TNF*, which are included in the Kaplan–Meier Plotter (https://tnmplot.com/analysis, accessed on 1 March 2024) [46]. We restricted our analysis to a specific type of tumor (e.g., testicular germ cell tumor, etc.) and considered all subtypes (stage, gender, grade, mutation, etc.) and cellular contents (basophils, B-cells, T-cells, macrophages, etc.).

### 2.10. Statistical Analysis

mRNA levels from the RT-qPCR results were normalized to the *EGFP* gene (5 × 10^−5^ pmol mixed to 10 μg of total RNA) to finally calculate the levels of each mRNA (×10^−6^ pmol) per 10 μg of total RNA. Values (n = 3) from the weeks of control (0) and Busulfan treatment (weeks 1–7) were continuously subjected to statistical analysis using SAS software version 9.2. All experimental data are presented as means ± SEMs. The significance (*p* < 0.05) among the means of the respective samples was determined using Duncan’s multiple range test as indicated by letters (a, b, c, d). The test was designed independently for each week, and mean values indicated with the same letter are not significantly different (ns).

## 3. Results

### 3.1. Busulfan Induces Apoptosis Signals and Reduces Spermatogenesis in Testicular Cells

**Figure 1:**
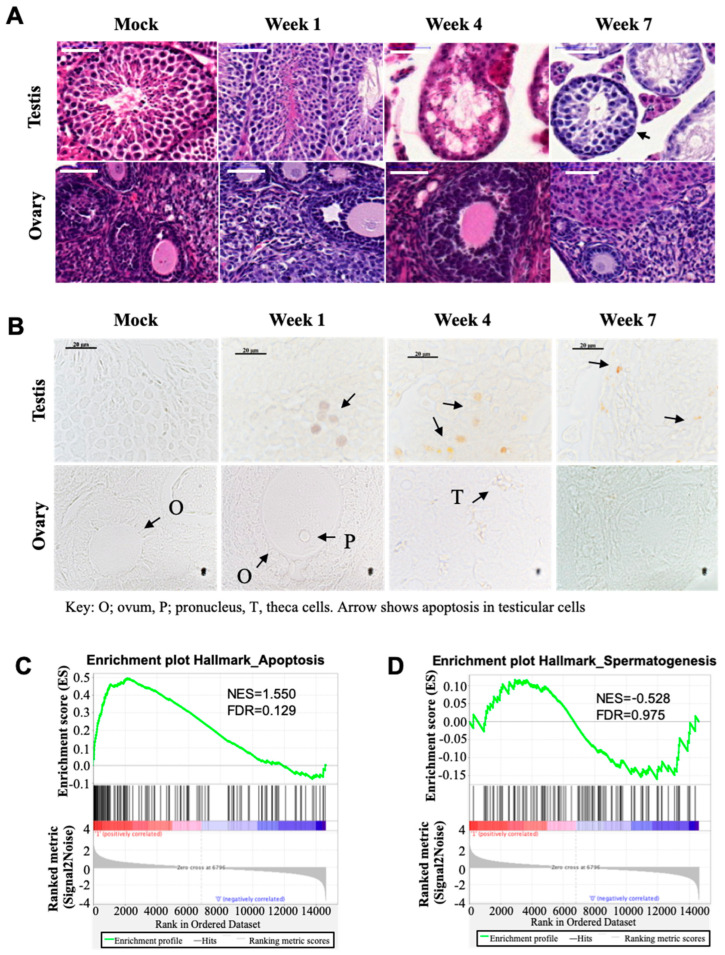
Busulfan reduces spermatogenesis and activates apoptosis signaling in the testis. (**A**) Photomicrographs of cross-sections from mouse testis and ovary. Tissue sections from 8-week-old DMSO-treated (mock) and Busulfan-treated mice were obtained after 1 week, 4 weeks, and 7 weeks of chemotherapy and stained in hematoxylin and eosin (H&E). Scale bar = 20 μm. (**B**) Apoptosis was determined using the TUNEL staining assay. Four weeks after Busulfan treatment, most testicular cells of mice were depleted except for a few somatic cells and spermatogonia. Arrowhead at week 7 in testis (panel A) represents new colonization of the seminiferous tubule. Apoptotic cells were identified in testicular cells, but there is no evidence of apoptosis in the ovary cell population. Arrows on the testis section (panel B) indicate apoptotic cells, stained brown. O; ovum, P; pronucleus, T, theca cells. Scale bar = 20 μm. (**C**,**D**) Gene set enrichment analysis (GSEA) of single cells from Busulfan-treated and DMSO-treated testis. The GSEA shows increased apoptotic signaling in spermatogonia stem cells (SSCs) (**C**) along with a reduction in spermatogenesis (**D**) in Busulfan-treated mice. The gene sets with FDR < 25% and nominal *p*-values <5% are considered significant.

To assess the side effects and response to Busulfan chemotherapy, we injected a single high dose of 40 mg/kg (body weight) of Busulfan in mice to induce the maximum number of apoptotic cells while minimizing the number of necrotic cells. Next, we excised the testis of male mice and the ovary of female mice (treated and not treated) for further H&E staining. Notably, we found that spermatogenic cells were significantly depleted in the Busulfan-treated testicular tissue after 4 weeks of chemotherapy, whereas a few spermatogonia and Sertoli cells remained attached to the lamina propria. The number of cells, such as somatic Leydig cells, in the intertubular space was also significantly decreased, but we detected new colonization at week 7 within testicular tubules and the intertubular space, suggesting that undifferentiated spermatogonia stem cells were relatively more resistant to carcinogens than differentiated spermatogonia. By contrast, we could not find any significant histological changes in size, shape, and localization of ovarian cells, including oocytes, granulosa, and stromal cells, in Busulfan-treated mice compared to the control (Figure 1A). To assess the apoptotic cells in the testicular and ovarian in response to Busulfan therapy, we performed a TUNEL staining assay. We found that Busulfan could induce the highest number of apoptotic cells in the testis. Interestingly, haploid germ cells were much more susceptible to apoptosis and were completely arrested at the crisis point of week 4. In the ovary, no noticeable apoptosis of ovarian cells was detected, suggesting the premeiotic spermatocytes in testicular cells likely underwent significant drug-induced cell arrest (Figure 1B). We further executed a gene set enrichment analysis (GSEA), which is a computational method to identify classes of genes that are over-represented in a large set of genes and may have an association with different phenotypes. We found increased apoptotic signaling in the single cells isolated from Busulfan-treated testis, accompanied by a reduction in the hallmarks of spermatogenesis (Figure 1C,D).

### 3.2. Busulfan Activates Oxidative Stress, DNA Repair, and Inflammatory Responses in Testicular Cells

**Figure 2:**
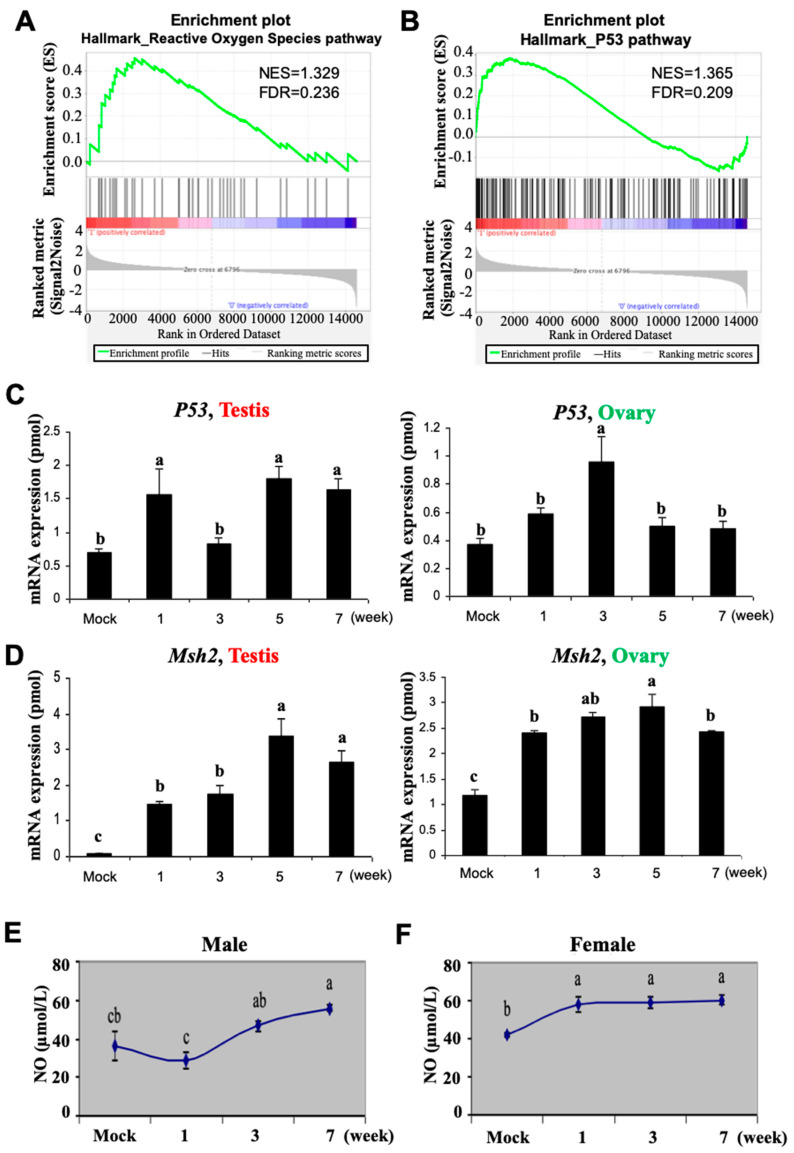
Busulfan activates oxidative stress followed by DNA repair signaling in the testis and ovary. Gene set enrichment analysis (GSEA) of single cells from Busulfan-treated mice shows enrichment of the reactive oxygen species (ROS) pathway (**A**) and the p53 signaling pathway (**B**) in spermatogonia stem cells (SSCs). The gene sets with FDR < 25% and nominal *p*-values < 5% are considered significant. (**C**,**D**) Quantification of mRNA amounts of biomarker genes, *p53*, and *Msh2*, in the testis and ovary. Data are from DMSO-treated samples (Mock) and Busulfan-treated tissues during weeks 1–7 of the experiment, as indicated on the X-axis. The amount of each mRNA (× 10^−6^ pmol) per 10 μg of total RNA is represented by the Y-axis. (**E**,**F**) Amount of NO in the serum male (**E**) and female (**F**) mice, where NO levels increased significantly after chemotherapy (μmol/L). The week of the treatment is shown on the *X*-axis. Bars indicate mean values; error bars indicate standard errors. Bar superscripts with at least one common letter (a, b, and c) are not significantly different at the 0.05 level (*p* < 0.05), according to Duncan’s multiple range test.

Reactive oxygen species (ROS) and the tumor suppressor p53 have a key function in regulating the apoptosis signaling pathway [47,48,49,50]. p53 also has a significant role in the DNA damage response [51]. Hence, we investigated whether Busulfan-induced apoptosis in testicular cells may be affected by ROS production and the activation of the p53 signaling pathway. Notably, the gene enrichment analysis of testicular cells revealed increased ROS production along with enrichment of the p53 signaling pathway in Busulfan-treated mice (Figure 2A,B). To validate which molecular signaling pathways are sensitive to Busulfan, the expression of several biomarker genes (Supplementary Table S1) in the mRNA samples collected from the testis versus ovary was analyzed using real-time PCR. We found that according to the mRNA levels, *TP53* is highly expressed in the male gonad, in contrast to the ovary, after 1 week of Busulfan treatment (Figure 2C). The mRNA levels of genes for DNA mismatch repair, *Msh2*, and excision repair, *Ercc1*, were significantly upregulated in both male and female gonads (*p* < 0.05) (Figure 2C). Interestingly, *Ercc5* expression was not significantly altered in the testis but slightly higher in the ovary (Supplementary Figure S2).

Furthermore, *NF-κB* and *Tgfβ1*, as regulators of immune tolerance [52,53], were also overexpressed during treatment (Supplementary Figure S2). From week 1, the expression of the *c-Kit* gene, a biomarker for the growth of oocytes and granulosa cells, was rapidly elevated in the ovary, similarly to the expression of *CDK4* and *Rb* cell cycle regulators, which were upregulated in both male and female gonads. We further checked the levels of nitric oxide (NO), as another free radical molecule [54,55], to measure the level of oxidative stress. We found that the production of NO was significantly elevated in the serum of Busulfan-treated males and females during all weeks of drug treatment, confirming the occurrence of global genotoxicity, leading to oxidative stress, inflammation, and DNA damage in this experimental setting.

### 3.3. Busulfan Downregulates the Expression of TAF7 and TNF in Testicular Cells

**Figure 3:**
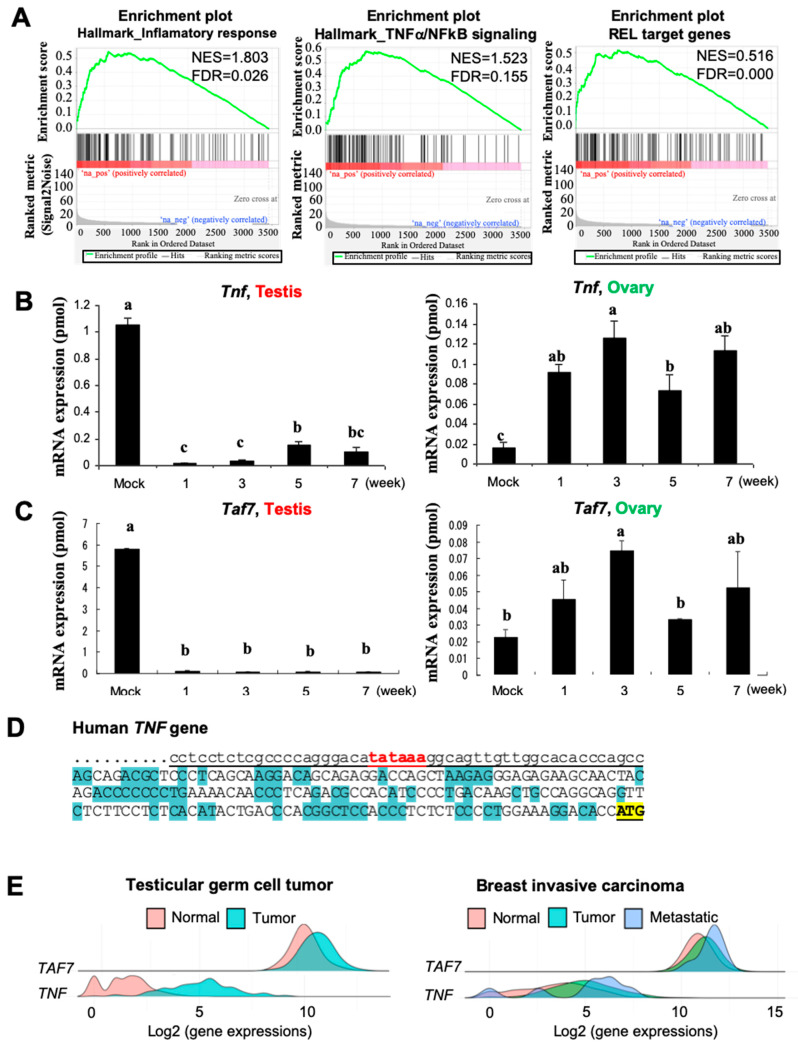
TAF7 together with TNF may regulate inflammatory signals in Busulfan-treated mice. (**A**) Gene set enrichment analysis (GSEA) of single cells from Busulfan-treated mice reveals a significant reduction in inflammatory responses, including TNF-α/NF-κβ signaling, via downregulation of REL-target genes in spermatogonia stem cells (SSCs). The gene sets with FDR < 25% and nominal p-values < 5% are considered significant. (**B**,**C**) Quantification of mRNA amounts of *TNF* and *TAF7* in testis and ovary. Data are from control samples (0 weeks) and Busulfan-treated tissues during weeks 1–7 of the experiment, as indicated on the X-axis. The amount of each mRNA (×10^−6^ pmol) per 10 μg of total RNA is represented by the Y-axis. Bars indicate mean values; error bars indicate standard errors. Bar superscripts with at least one common letter (a, b, and c) are not significantly different at the 0.05 level (*p* < 0.05), according to Duncan’s multiple range test. (**D**) Alignment of promoter sequences from the human TNF gene (ID: 7124) demonstrates the TATA box-binding motif (red) may bind to the TAF7 protein. The underlined DNA sequence is the promoter of the *TNF* gene. (**E**) Differential gene expression analysis of *TAF7* and *TNF* was performed using RNA-Seq data from testicular germ cell tumor (left) and breast invasive carcinoma (right). The expression of *TAF7* and *TNF* genes as a density plot comparing normal, tumor, and metastatic tissues.

From the single-cell analysis of testicular cells using GSEA, we found that inflammation responses occur through the TNF-α/NF-kB signaling pathway, which is reduced after a single dose of Busulfan treatment, followed by downregulation of REL-target genes (Figure 3A). Through the screening and evaluation of the mRNA levels of several biomarkers, we determined that *TNF* and *TAF7* were significantly downregulated in testis after Busulfan treatment compared to untreated mice (Figure 3B,C). Interestingly, the expression of both *TNF* and *TAF7* were upregulated in the ovary after treatment during weeks 1–7 of the experiment. We also confirmed the reduction in the levels of TAF7 and TNF protein expression during Busulfan treatment (1–7 weeks) using a Western blotting analysis (Supplementary Figure S3A). Notably, TAF7 is a TATA box-binding protein that binds to the promoter of several genes to recruit RNA polymerase II for activation of transcription [35]. Indeed, the alignment of promoter sequences from the human TNF gene (ID: 7124) and mouse TNF gene (ID: 21926) uncovered the TATA box-binding motif for possible binding with the TAF7 protein in both species (Figure 3D, Supplementary Figure S3B).

**Figure 4:**
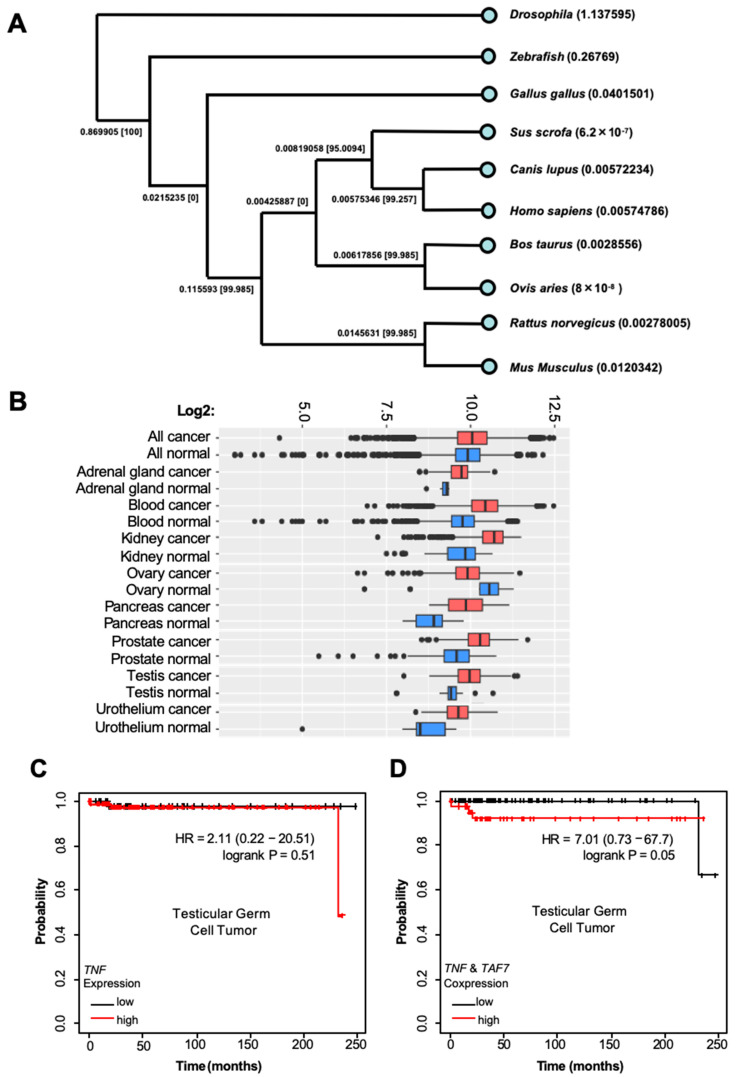
TAF7 is highly conserved within species and expressed in several types of human cancers with poor survival outcomes. (**A**) Phylogenetic analysis of TAF7-amino acid sequences in several species such as *Homo sapiens* (human), *Canis lupus* (dog), *Sus scrofa* (pig), *Bos taurus* (cow), *Ovis aries* (sheep), *Gallus gallus* (chicken), zebrafish, and *Drosophila*. (**B**) mRNA expression pattern of 34,000 samples was analyzed and profiled using Affymetrix U133A platforms. Box shows TAF7 overexpression in blood, kidney, pancreas, prostate, and testis cancers. Interestingly, the mRNA level of *TAF7* is downregulated in ovary cancer. N, normal (green) versus C, cancer (red). (**C**,**D**) The Kaplan–Meier survival curve for patients with testicular germ cell tumor (n = 134) with low and high expression of *TAF7* or *TNF*, respectively ((**C**), survival probability: HR = 7.01 (0.73–67.7); log-rank *p* = 0.05), and low and high co-expression of *TAF7* and *TNF* ((**D**), survival probability: HR = 7.01 (0.73–67.7); log-rank *p* = 0.05).

In addition, we performed a differential gene expression analysis of *TAF7* and *TNF* using RNA-Seq-based data [45] from testicular germ cell tumors and breast invasive carcinoma. We found that the gene expression of *TAF7* and *TNF* is induced upon tumorigenesis and more greatly overexpressed in response to the metastatic level of breast invasive carcinoma (Figure 3E). The genomic structure of *TAF7* also reveals a distinct difference in the number of coding and non-coding sequences of exons compared to TAF7l (TATA box-binding protein-associated factor 7-like) (Supplementary Figure S4A), which can be replaced by TAF7 as a TFIID subunit in late pachytene spermatocytes and haploid cells [56]. This suggests the unique function of TAF7, rather than TAF7l, in the regulation of transcriptional initiation. We further performed a multiple sequence alignment and phylogenetic analysis of TAF7 at the protein level and found that the amino acid sequence of human TAF7 is conserved and more highly similar to the corresponding sequences in *Canis lupus* (dog) and *Sus scrofa* (swine) than those in *Bos taurus* (cow), *Ovis aries* (lamb), and *Mus musculus* (mouse) (Figure 4A).

To specify whether *TAF7* is highly expressed in cancer and regulates tumorigenesis, we analyzed the gene expression database of normal and tumor tissues of the National Genomics Data Center and found that *TAF7* is more differentially expressed in blood, kidney, pancreas, prostate, and testis cancers compared to the corresponding normal tissues (Figure 4B). Lastly, an analysis of patient survival with testicular germ cell tumors along with other types of cancers revealed high expression levels of both *TAF7* and *TNF*, which were strongly associated with poor survival outcomes in patients (Figure 4C,D, Supplementary Figure S4B–E). Indeed, a low and high expression of *TAF7* and *TNF* in testicular germ cell tumors together could drive poor survival outcomes in patients, while a low and high expression of *TAF7* or *TNF* in tumors alone is associated with extended longevity. These findings may be important for the prognostic and therapeutic approaches used for cancer patients with the expression of *TAF7* and *TNF* in testicular germ cells.

### 3.4. TAF7 Is Localized in Spermatogonia Stem Cells and Expressed at Lower Levels in Female Oocytes

**Figure 5:**
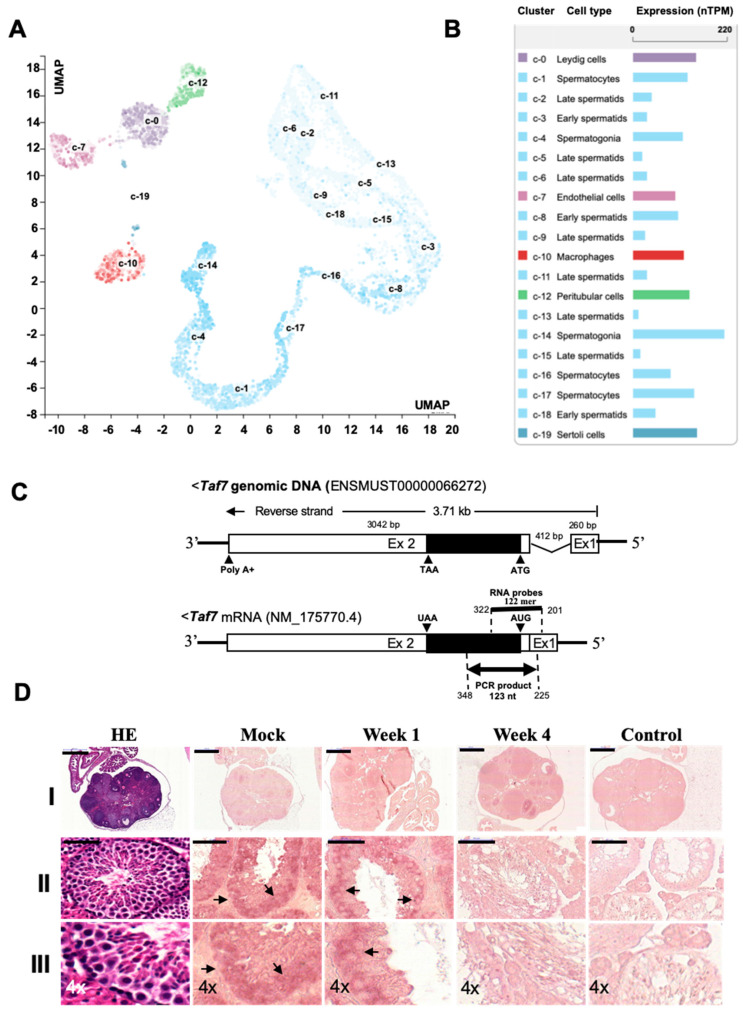
TAF7 is highly expressed and localized in the male germ cells. (**A**,**B**) RNA expression of *TAF7* in single-cell type clusters was identified in testis and visualized as a UMAP plot (**A**) and a bar chart (**B**). UMAP plot showing the cells in each cluster (c-0 to c-19), where each dot corresponds to a cell (**A**), and the name of each cluster is highlighted in the bar chart (**B**). The bar chart shows the value for *TAF7* RNA expression (nTPM) in each cell type cluster. (**C**) The genomic sequence and mRNA of the *TAF7* gene. Target sequences for real-time PCR and RNA probes in *TAF7* mRNA are shown. Coding/non-coding sequences of exons are shown in black and white boxes, respectively, and the exon number is indicated inside the boxes. Positions of fragments amplified in real-time PCR (123 bp) are indicated by horizontal bi-directional arrows, while those of sense/antisense cRNA probes (122-mer) are indicated by horizontal bold lines. (**D**) Localization and expression level of *TAF7* sense transcripts in ovary and testis. *TAF7* was localized and expressed in testicular germ cells (lines II and III) as examined by in situ hybridization and showed by the arrows. There are not any significant changes in *TAF7* expression in the ovary (top Line I). Sections were obtained from DMSO-treated (Mock), and Busulfan-treated testis after 1 and 4 weeks of treatment. Scale bars are 100 μm for panel I and 20 μm for panel II, which are enlarged with 4× magnification in panel III. Control: A 120 nt lambda phage sequence was used as a negative control RNA probe.

We further intended to specify the cell types where *TAF7* might be localized and expressed for testicular and ovarian cell growth. We performed a single-cell type analysis and clustering of RNA-Seq data, publicly available in the GEO. We visualized the cells with *TAF7* expression in each cluster using a UMAP plot along with the intensity of this expression in the bar chart. To this end, the expression data within cell type clusters are assembled to generate a normalized transcript per million (nTPM) for each gene and cell type [57] (Figure 5A,B). We found that based on mRNA levels, *TAF7* is highly expressed in spermatogonia (213.4 nTPM), spermatocytes (143.1 nTPM), Leydig cells (146.5 nTPM), Sertoli cells (143.6 nTPM), peritubular cells (132.2 nTPM), and macrophages (117.8 nTPM). However, the mRNA level of *TAF7* is lower in early and late spermatids (approximately around 32 and 44 nTPM) and endothelial cells (98.6 nTPM) (Figure 5A,B). In addition, *TAF7* is highly expressed in ovarian stromal, endothelial, and smooth muscle cells, especially in granulosa cells (202.6 nTPM), while it is expressed at lower levels in the oocyte, as a female germ cell (27.9 nTPM) (Supplementary Figure S5A,B). We further validated the expression of single-cell RNA-Seq data using in situ hybridization (ISH) [40,41]. We designed 122-mer DIG-labeled RNA probes to be hybridized with the sense transcript of *TAF7* mRNA, which is shown in the schematic images of genomic DNA and mRNA structure of *TAF7* (Figure 5C). Indeed, using ISH, high levels of *TAF7* sense transcripts were detected in the testis but not in the ovary, both before and after Busulfan treatment. In DMSO-treated testis, most of the *TAF7* sense transcript was localized in the basal compartment, where the spermatogonia stem cells are located. Furthermore, the signal intensity of *TAF7* sense transcripts was profoundly decreased in the testis after one week of treatment (Figure 5D, lines II-III). In the ovary, the sense transcript of *TAF7* was not detected in oocytes or theca folliculi, except in granulosa cells and the corpus luteum (Figure 5D, lines I), which was predicted based on the results from single-cell RNA-Seq data (Figure 5A,B). No hybridization-based interactions were detected using negative control probes (a 120 nt lambda phage sequence) in tissues. These results are consistent with the data obtained from the quantitative RT-PCR and Western blotting analysis, suggesting that *TAF7* expression is reduced upon drug treatment in testis. Thus, the localization and expression of *TAF7* in spermatogonia may suggest a possible function of *TAF7* in spermiogenesis, which is downregulated upon Busulfan treatment.

### 3.5. Busulfan Upregulates the Inflammatory Response for Downregulation of Testosterone in Sertoli Cells

**Figure 6:**
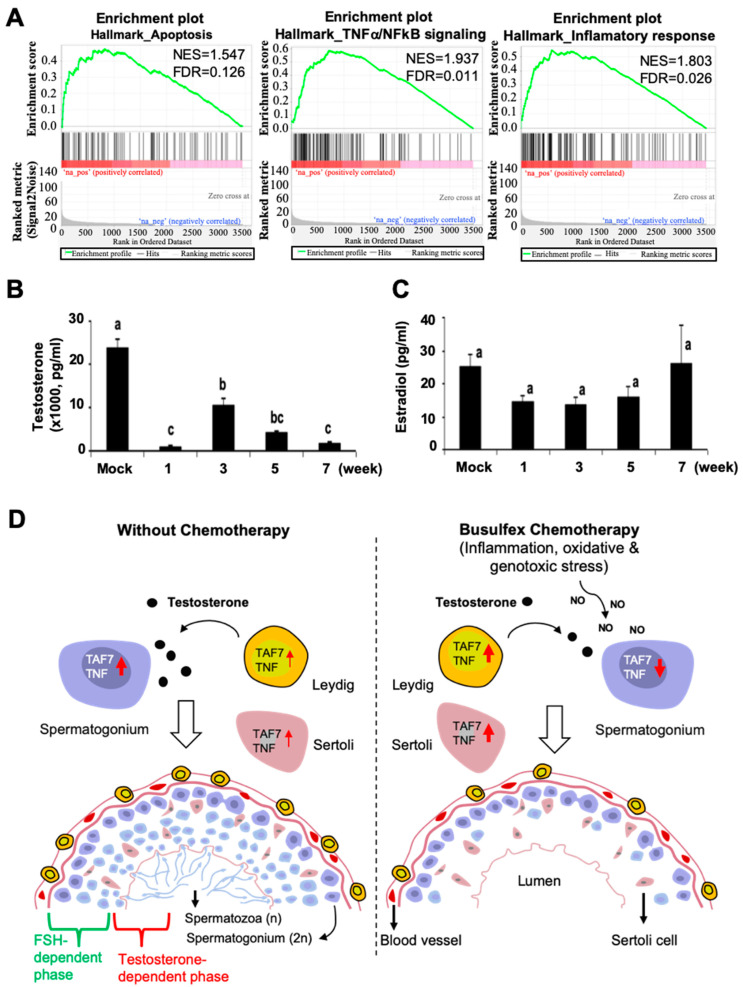
Busulfan reduces serum testosterone and induces apoptosis and inflammatory responses in Sertoli cells. (**A**) Gene set enrichment analysis (GSEA) of single cells from Busulfan-treated mice shows elevated levels of apoptosis, TNF-α/NF-kB signaling, and inflammatory responses in Sertoli cells. The gene sets with FDR < 25% and nominal *p*-values < 5% are considered significant. (**B**,**C**) Enzyme immunoassay (EIA) to detect testosterone (**B**) and estradiol (**C**) levels in the serum of male and female mice. Testosterone was significantly decreased after drug treatment during the weeks of the experiment, but estradiol remained unchanged at all times. The week of the experiment is represented by the X-axis. Bars indicate mean values; error bars are the standard error of the mean. Bar superscripts (a, b, and c) with different letters indicate significance (*p* < 0.05) regarding differences between the means of the respective samples, according to Duncan’s multiple range test. (**D**) A schematic representation of the possible mechanism for male germ cell dysfunction after Busulfan chemotherapy. Busulfan induces apoptosis and may trigger several cellular signaling events in spermatogonia stem cells, spermatocytes, and somatic cells (e.g., Sertoli, Leydig). This may be due to several reasons: (1) Steady-state *TAF7* expression may regulate the initiation of *TNF* transcription. Herein, binding of TAF7 to the TATA box on the *TNF* promoter may lead to spermatogonia stem cell survival and colonization (Left). However, Busulfan may interfere with DNA to dissociate TAF7 binding and repress *TNF* expression, leading to apoptosis induction in spermatogonia stem cells (Right). (2) Genes for DNA repair signaling (e.g., *Ercc1*, *Msh2*) are highly expressed in response to genotoxic and oxidative stress (in both male and female gonads); however, downregulation of *TAF7* in testis might initiate premature transcription through RNA polymerase II for spermatogonia stem cell arrest. (3) Busulfan promotes DNA–DNA interstrand crosslinking between DNA bases, which cannot be repaired by the cellular machinery, and the cells undergo apoptosis. (4) Busulfan treatment activates the TNF-α/NF-kB signaling pathway for the inflammatory response, which inhibits the production of testosterone, leading to male infertility.

We demonstrated that *TAF7* is highly expressed in spermatogonia germ cells and spermatocytes. We subsequently performed a GSEA to reveal which somatic cell types have an enriched TAF7 transcriptome in response to Busulfan. We revealed that the RNA sequence of the *TAF7* transcript is gradually enriched in Sertoli cells, which are responsible for the progression of germ cells to spermatozoa within the seminiferous tubules [58,59]. Moreover, the levels of apoptosis signaling, TNF-α/NF-kB signaling pathway activity, and inflammatory responses were increased in Sertoli cells in response to drug treatment (Figure 6A). Furthermore, we analyzed testosterone and estradiol levels to trace steroid-related hormonal responses in males and females. In our data, the level of testosterone, which is produced by Leydig cells, was significantly (*p* < 0.05) diminished in male serum after Busulfan treatment, which may increase the susceptibility of testicular cells to apoptosis (Figure 6B). Moreover, the measurements showed that estradiol, which is synthesized in the ovarian granulosa cells and placenta, did not significantly change during treatment (Figure 6B). This confirms that activation of the TNF-α/NF-kB signaling pathway may downregulate testosterone production in Leydig cells, leading to male infertility. However, ovarian cells remained healthy, with normal levels of estradiol in female serum that remained unchanged upon treatment. The range of the standard curve was determined to be 1017–23,833 pg/mL for testosterone and 13.42–26.1 pg/mL for estradiol compared to the control sample during the weeks of treatment.

## 4. Discussion

Chemotherapy with alkylating agents may trigger several cellular signaling pathways in both germ and somatic cells. An immediate result of treatment using Busulfan is the induction of apoptosis in spermatogonia stem cells and spermatocytes [60]. In contrast, a small subpopulation of spermatogonia is attached to the basal membrane of the seminiferous epithelium and maintained for recolonization [61,62,63]. In females, apoptosis is reported to be induced in oocytes in response to proapoptotic factors, thereby affecting oogenesis and folliculogenesis [64,65]. Consistently, we revealed that there is reduced spermatogenesis in the seminiferous tubules and also increased apoptosis in the testis. However, we could not determine any significant level of apoptosis signals in the ovary. This may be due to the different concentrations of drugs and strains of mice used for the treatments. Indeed, several pro-survival and proapoptotic factors are involved in testicular and ovarian apoptosis; therefore, their balance may be disrupted in response to different stimuli and factors.

Busulfan, as an alkylsulfonate agent, promotes DNA–DNA interstrand crosslinking between DNA bases [66], which cannot be repaired by the cellular machinery, and the cells undergo apoptosis [67]. Notably, DNA crosslinking can prevent DNA replication [68,69], leading to cell arrest. To this end, nucleotide excision repair (NER), which includes the global genomic repair (GGR) and transcription-coupled repair (TCR) sub-pathways, provides an important cellular defense against structurally unrelated alterations in DNA [70,71,72,73]. Most of these alterations, if unrepaired, may contribute to mutagenesis, oncogenesis, and developmental abnormalities, as well as cell death [74]. Herein, we found that DNA excision repair (*Ercc1*) and mismatch repair (*Msh2*) genes are highly expressed after Busulfan treatment (Figure 2). This indicates that nuclear excision repair (NER) is activated in response to genotoxic stress in both sexes. However, the initiation of premature transcription may be activated by RNA polymerase II after chemotherapy in testis. This is partly due to TAF7, functioning as a checkpoint regulator, which inhibits premature transcription initiation until the assembly of the preinitiation complex is complete [75]. This might provide important signals for apoptosis to be triggered in viable cells or for their shift into abnormal development, leading to dysregulated spermatogenesis and infertility.

It is shown that the biological function of TNF and NF-κB are closely associated and their signaling pathways undergo cross-talk within numerous tissues [76,77]. Our data show that *TAF7* is highly expressed in the testis and to a lesser extent in the ovary. Moreover, it is downregulated in the testis upon Busulfan treatment (*p* < 0.05; Figure 3C). Notably, *TNF* is also highly expressed in DMSO-treated testis, but its expression decreases earlier in the testis in response to Busulfan treatment. Consistently, the GSEA of single-cell RNA-Seq data shows that inflammatory responses are activated through the TNF-α/NF-kB signaling pathway for the upregulation of REL-target genes (Figure 3A). Therefore, this inflammatory response can destroy reproductive cells and tissues such as sperm, testis, and eggs in the ovaries. In addition, it can activate an autoimmune response, driving the body to attack reproductive tissues. In our experiment settings, *TAF7* and *TNF* were highly expressed in the testis and ovary; however, they were downregulated in the testis but not in the ovary in response to Busulfan treatment. This may be due to their specific function in the regulation of spermatogenesis. We further checked their association with the regulation of tumor cell survival and cancer progression, finding that *TAF7* is highly expressed in several cancers in comparison to normal tissues (Figure 4B). Interestingly, testicular germ cell tumor patients co-expressing *TNF* and *TAF7* had poorer survival outcomes compared to those with expression of *TNF* alone (Figure 3E and Supplementary Figure S4B–E). Indeed, we found a TATA box-binding motif on the promoter of human and mouse *TNF* genes, which might facilitate binding with TAF7 for transcription initiation (Figure 3D and Supplementary Figure S3B). However, support for this claim requires more in-depth studies involving ChIP-seq analysis. Therefore, TAF7 may have an antiapoptotic function, given that spermatogonia stem cells with high amounts of *TAF7* are more resistant to Busulfan treatment, maintaining self-renewal, proliferation, and colonization. Indeed, silencing *TAF7* in clear cell renal cell carcinoma (ccRCC) cells inhibits their proliferation, reduces migration, and promotes apoptosis, further supporting the role of TAF7 in cell survival mechanisms [39].

We further conducted an enrichment analysis of *TAF7* expression, which was followed by ISH analysis for the confirmation of *TAF7* localization in testicular cells. We found that *TAF7* is highly expressed and localized in spermatogonia stem cells, spermatocytes, and especially in the early spermatids (Figure 5). We could also determine the expression of *TAF7* in endothelial, peritubular, Leydig, and Sertoli cells along with macrophages (Figure 5A). However, a lower *TAF7* expression was detected in female germ cells (Supplementary Figure S5). Notably, *TNF* is responsible for inflammatory responses [25,78] and is also highly expressed in Leydig and Sertoli cells [79]. In our experiment settings, the enrichment of apoptosis and inflammatory responses by the TNF-α/NF-kB signaling pathway peaked, and then the amount of *TNF* mRNA sharply and significantly decreased after 1 week of drug treatment. This result was followed by a reduction in the testosterone levels in male serum, while the level of estradiol remained consistent during treatment. In fact, NF-kB DNA-binding activity is significantly suppressed by testosterone [80,81,82]. This suggests that physiological testosterone concentrations may exert their anti-inflammation effects on TAF7/TNF-α signaling in the testis. When testosterone levels are reduced in response to drug treatment, the extracellular secretion of TNF is activated in Leydig and Sertoli cells, which in turn suppresses intracellular TAF7/TNF-α signaling in the spermatogonium as part of a negative feedback loop. Support for this concept requires further study based on measuring the cytokines produced by testicular cells with *TAF7* or *TNF* shRNA knockdowns cultured in a 3D system.

## 5. Conclusions

We showed that male germinal tissue is more susceptible to chemotherapy than female gonad tissue; therefore, the renewal of DNA repair and abolishing inflammatory responses may be critical for restoring fertility in mice. We postulate that the early loss of testosterone, *TAF7,* and *TNF* may cause infertility in males following Busulfan genotoxicity in the testis. Since Busulfan chemotherapy mediates male germ cell dysfunction, we recommend using monoclonal antibodies in combination with lower amounts of Busulfan to inhibit TNF function. The results suggest that efficiently changing regimens in male versus female treatment in cancer therapy may also be important. However, a significant amount of research is still needed to identify whether key markers of SSCs, such as TAF7 and TNF, could be associated with the impact of chemotherapy drugs and can thus be targeted for preserving spermatogonia stem cell populations following Busulfan chemotherapy.

## Data Availability

Data are contained within the article.

## Supplementary Materials

The following supporting information can be downloaded at: https://www.mdpi.com/article/10.3390/biomedicines12102220/s1, Figure S1: Busulfan reduces spermatogenesis and activates apoptosis signaling in the testis; Figure S2: Busulfan activates DNA repair signaling in the testis and ovary; Figure S3: Busulfan may downregulate *TAF7* for the reduction in the level of *TNF* expression; Figure S4: *TAF7* is highly expressed in several types of human cancers with poor survival outcomes; Figure S5: *TAF7* is not highly expressed in female germ cells; Table S1: Primer sequences for qRT-PCR; Table S2: Taf7 probe sequence.

## References

[1] Schiffman K.S., Bensinger W.I., Appelbaum F.R., Rowley S., Lilleby K., Clift R.A., Weaver C.H., Demirer T., Sanders J.E., Petersdorf S. (1996). Phase II study of high-dose busulfan, melphalan and thiotepa with autologous peripheral blood stem cell support in patients with malignant disease. Bone Marrow Transplant..

[2] Santos F.P., Kantarjian H., Quintas-Cardama A., Cortes J. (2011). Evolution of therapies for chronic myelogenous leukemia. Cancer J..

[3] Cheng F., Yuan G., Li Q., Cui Z., Li W. (2023). Long-term outcomes of frontline imatinib therapy for chronic myeloid leukemia in China. Front. Oncol..

[4] Lesurtel M., Graf R., Aleil B., Walther D.J., Tian Y., Jochum W., Gachet C., Bader M., Clavien P.A. (2006). Platelet-derived serotonin mediates liver regeneration. Science.

[5] Brisse H., Orbach D., Lassau N., Servois V., Doz F., Debray D., Helfre S., Hartmann O., Neuenschwander S. (2004). Portal vein thrombosis during antineoplastic chemotherapy in children: Report of five cases and review of the literature. Eur. J. Cancer.

[6] Grigg A., Gibson R., Bardy P., Szer J. (1996). Acute portal vein thrombosis after autologous stem cell transplantation. Bone Marrow Transplant..

[7] Jain R., Gupta K., Bhatia A., Bansal A., Bansal D. (2017). Hepatic Sinusoidal-obstruction Syndrome and Busulfan-induced Lung Injury in a Post-autologous Stem Cell Transplant Recipient. Indian. Pediatr..

[8] Poorvu P.D., Frazier A.L., Feraco A.M., Manley P.E., Ginsburg E.S., Laufer M.R., LaCasce A.S., Diller L.R., Partridge A.H. (2019). Cancer Treatment-Related Infertility: A Critical Review of the Evidence. JNCI Cancer Spectr..

[9] Gosden R., Spears N. (1997). Programmed cell death in the reproductive system. Br. Med. Bull..

[10] Lewis-Wambi J.S., Jordan V.C. (2009). Estrogen regulation of apoptosis: How can one hormone stimulate and inhibit?. Breast Cancer Res..

[11] Li D., Chen J., Ai Y., Gu X., Li L., Che D., Jiang Z., Li L., Chen S., Huang H. (2019). Estrogen-Related Hormones Induce Apoptosis by Stabilizing Schlafen-12 Protein Turnover. Mol. Cell.

[12] Zohni K., Zhang X., Tan S.L., Chan P., Nagano M.C. (2012). The efficiency of male fertility restoration is dependent on the recovery kinetics of spermatogonial stem cells after cytotoxic treatment with busulfan in mice. Hum. Reprod..

[13] Kerbauy M.N., Mariano L., Seber A., Rocha V. (2020). The impact of low dose busulfan on gonodal function after allogeneic hematopoietic stem cell transplantation for aplastic anemia. Bone Marrow Transplant..

[14] Mobarak H., Rahbarghazi R., Nouri M., Heidarpour M., Mahdipour M. (2022). Intratesticular versus intraperitoneal injection of Busulfan for the induction of azoospermia in a rat model. BMC Pharmacol. Toxicol..

[15] Wang X., Lin Y. (2008). Tumor necrosis factor and cancer, buddies or foes?. Acta Pharmacol. Sin..

[16] Sethi J.K., Hotamisligil G.S. (2021). Metabolic Messengers: Tumour necrosis factor. Nat. Metab..

[17] Oeckinghaus A., Ghosh S. (2009). The NF-kappaB family of transcription factors and its regulation. Cold Spring Harb. Perspect. Biol..

[18] Capece D., Verzella D., Flati I., Arboretto P., Cornice J., Franzoso G. (2022). NF-kappaB: Blending metabolism, immunity, and inflammation. Trends Immunol..

[19] Guo Q., Jin Y., Chen X., Ye X., Shen X., Lin M., Zeng C., Zhou T., Zhang J. (2024). NF-kappaB in biology and targeted therapy: New insights and translational implications. Signal Transduct. Target. Ther..

[20] Parameswaran N., Patial S. (2010). Tumor necrosis factor-alpha signaling in macrophages. Crit. Rev. Eukaryot. Gene Expr..

[21] Siegmund D., Wajant H. (2023). TNF and TNF receptors as therapeutic targets for rheumatic diseases and beyond. Nat. Rev. Rheumatol..

[22] Chun N., Ang R.L., Chan M., Fairchild R.L., Baldwin W.M., Horwitz J.K., Gelles J.D., Chipuk J.E., Kelliher M.A., Pavlov V.I. (2021). T cell-derived tumor necrosis factor induces cytotoxicity by activating RIPK1-dependent target cell death. JCI Insight.

[23] Wajant H., Siegmund D. (2019). TNFR1 and TNFR2 in the Control of the Life and Death Balance of Macrophages. Front. Cell Dev. Biol..

[24] Yang S., Xie C., Chen Y., Wang J., Chen X., Lu Z., June R.R., Zheng S.G. (2019). Differential roles of TNFalpha-TNFR1 and TNFalpha-TNFR2 in the differentiation and function of CD4(+)Foxp3(+) induced Treg cells in vitro and in vivo periphery in autoimmune diseases. Cell Death Dis..

[25] van Loo G., Bertrand M.J.M. (2023). Death by TNF: A road to inflammation. Nat. Rev. Immunol..

[26] Yang P., McKay B.S., Allen J.B., Jaffe G.J. (2004). Effect of NF-kappa B inhibition on TNF-alpha-induced apoptosis in human RPE cells. Invest. Ophthalmol. Vis. Sci..

[27] Muralidharan S., Mandrekar P. (2013). Cellular stress response and innate immune signaling: Integrating pathways in host defense and inflammation. J. Leukoc. Biol..

[28] Jeong Y., Lim J.W., Kim H. (2019). Lycopene Inhibits Reactive Oxygen Species-Mediated NF-kappaB Signaling and Induces Apoptosis in Pancreatic Cancer Cells. Nutrients.

[29] Zhao J., Jiang P., Guo S., Schrodi S.J., He D. (2021). Apoptosis, Autophagy, NETosis, Necroptosis, and Pyroptosis Mediated Programmed Cell Death as Targets for Innovative Therapy in Rheumatoid Arthritis. Front. Immunol..

[30] Masli S., Turpie B. (2009). Anti-inflammatory effects of tumour necrosis factor (TNF)-alpha are mediated via TNF-R2 (p75) in tolerogenic transforming growth factor-beta-treated antigen-presenting cells. Immunology.

[31] Spender L.C., O’Brien D.I., Simpson D., Dutt D., Gregory C.D., Allday M.J., Clark L.J., Inman G.J. (2009). TGF-beta induces apoptosis in human B cells by transcriptional regulation of BIK and BCL-XL. Cell Death Differ..

[32] Kawabata K.C., Ehata S., Komuro A., Takeuchi K., Miyazono K. (2013). TGF-beta-induced apoptosis of B-cell lymphoma Ramos cells through reduction of MS4A1/CD20. Oncogene.

[33] Liu Z.W., Zhang Y.M., Zhang L.Y., Zhou T., Li Y.Y., Zhou G.C., Miao Z.M., Shang M., He J.P., Ding N. (2021). Duality of Interactions Between TGF-beta and TNF-alpha During Tumor Formation. Front. Immunol..

[34] Chiang C.M., Roeder R.G. (1995). Cloning of an intrinsic human TFIID subunit that interacts with multiple transcriptional activators. Science.

[35] Chen X., Qi Y., Wu Z., Wang X., Li J., Zhao D., Hou H., Li Y., Yu Z., Liu W. (2021). Structural insights into preinitiation complex assembly on core promoters. Science.

[36] Munz C., Psichari E., Mandilis D., Lavigne A.C., Spiliotaki M., Oehler T., Davidson I., Tora L., Angel P., Pintzas A. (2003). TAF7 (TAFII55) plays a role in the transcription activation by c-Jun. J. Biol. Chem..

[37] Pal S., Paladhi P., Dutta S., Bose G., Ghosh P., Chattopadhyay R., Chakravarty B., Saha I., Ghosh S. (2021). Novel variations in spermatogenic transcription regulators RFX2 and TAF7 increase risk of azoospermia. J. Assist. Reprod. Genet..

[38] Nakagawa T., Hosogane M., Nakagawa M., Morohoshi A., Funayama R., Nakayama K. (2018). Transforming Growth Factor beta-Induced Proliferative Arrest Mediated by TRIM26-Dependent TAF7 Degradation and Its Antagonism by MYC. Mol. Cell Biol..

[39] Zhang J., Duan B., Li F., Jing X., Li R., Cai S., Cao L., Jiang Q., Zhou J., Zhou J. (2024). SETD7 Promotes Cell Proliferation and Migration via Methylation-mediated TAF7 in Clear Cell Renal Cell Carcinoma. Int. J. Biol. Sci..

[40] Rezaeian A.H., Isokane T., Nishibori M., Chiba M., Hiraiwa N., Yoshizawa M., Yasue H. (2009). alphaCGRP and betaCGRP transcript amount in mouse tissues of various developmental stages and their tissue expression sites. Brain Dev..

[41] Rezaeian A.H., Katafuchi T., Yoshizawa M., Hiraiwa N., Saito T., Nishibori M., Hamano K., Minamino N., Yasue H. (2008). Genomic organization, expression and evolution of porcine CRSP1, 2, and 3. Cytogenet. Genome Res..

[42] Huerta-Cepas J., Serra F., Bork P. (2016). ETE 3: Reconstruction, Analysis, and Visualization of Phylogenomic Data. Mol. Biol. Evol..

[43] Gascuel O. (1997). BIONJ: An improved version of the NJ algorithm based on a simple model of sequence data. Mol. Biol. Evol..

[44] Guindon S., Dufayard J.F., Lefort V., Anisimova M., Hordijk W., Gascuel O. (2010). New algorithms and methods to estimate maximum-likelihood phylogenies: Assessing the performance of PhyML 3.0. Syst. Biol..

[45] Bartha A., Gyorffy B. (2021). TNMplot.com: A Web Tool for the Comparison of Gene Expression in Normal, Tumor and Metastatic Tissues. Int. J. Mol. Sci..

[46] Gyorffy B. (2024). Integrated analysis of public datasets for the discovery and validation of survival-associated genes in solid tumors. Innovation.

[47] Shen Y., White E. (2001). p53-dependent apoptosis pathways. Adv. Cancer Res..

[48] Simon H.U., Haj-Yehia A., Levi-Schaffer F. (2000). Role of reactive oxygen species (ROS) in apoptosis induction. Apoptosis.

[49] Liu Y., Su Z., Tavana O., Gu W. (2024). Understanding the complexity of p53 in a new era of tumor suppression. Cancer Cell.

[50] Sies H., Belousov V.V., Chandel N.S., Davies M.J., Jones D.P., Mann G.E., Murphy M.P., Yamamoto M., Winterbourn C. (2022). Defining roles of specific reactive oxygen species (ROS) in cell biology and physiology. Nat. Rev. Mol. Cell Biol..

[51] Rodriguez-Pastrana I., Birli E., Coutts A.S. (2023). p53-dependent DNA repair during the DNA damage response requires actin nucleation by JMY. Cell Death Differ..

[52] Barnabei L., Laplantine E., Mbongo W., Rieux-Laucat F., Weil R. (2021). NF-kappaB: At the Borders of Autoimmunity and Inflammation. Front. Immunol..

[53] Yang D., Dai F., Yuan M., Zheng Y., Liu S., Deng Z., Tan W., Chen L., Zhang Q., Zhao X. (2021). Role of Transforming Growth Factor-beta1 in Regulating Fetal-Maternal Immune Tolerance in Normal and Pathological Pregnancy. Front. Immunol..

[54] Xu W., Liu L.Z., Loizidou M., Ahmed M., Charles I.G. (2002). The role of nitric oxide in cancer. Cell Res..

[55] Lundberg J.O., Weitzberg E. (2022). Nitric oxide signaling in health and disease. Cell.

[56] Pointud J.C., Mengus G., Brancorsini S., Monaco L., Parvinen M., Sassone-Corsi P., Davidson I. (2003). The intracellular localisation of TAF7L, a paralogue of transcription factor TFIID subunit TAF7, is developmentally regulated during male germ-cell differentiation. J. Cell Sci..

[57] Digre A., Lindskog C. (2023). The human protein atlas-Integrated omics for single cell mapping of the human proteome. Protein Sci..

[58] Griswold M.D. (1998). The central role of Sertoli cells in spermatogenesis. Semin. Cell Dev. Biol..

[59] O’Donnell L., Smith L.B., Rebourcet D. (2022). Sertoli cells as key drivers of testis function. Semin. Cell Dev. Biol..

[60] Choi Y.J., Ok D.W., Kwon D.N., Chung J.I., Kim H.C., Yeo S.M., Kim T., Seo H.G., Kim J.H. (2004). Murine male germ cell apoptosis induced by busulfan treatment correlates with loss of c-kit-expression in a Fas/FasL- and p53-independent manner. FEBS Lett..

[61] Anand S., Bhartiya D., Sriraman K., Mallick A. (2016). Underlying Mechanisms that Restore Spermatogenesis on Transplanting Healthy Niche Cells in Busulphan Treated Mouse Testis. Stem Cell Rev. Rep..

[62] Kanatsu-Shinohara M., Toyokuni S., Morimoto T., Matsui S., Honjo T., Shinohara T. (2003). Functional assessment of self-renewal activity of male germline stem cells following cytotoxic damage and serial transplantation. Biol. Reprod..

[63] La H.M., Liao J., Legrand J.M.D., Rossello F.J., Chan A.L., Vaghjiani V., Cain J.E., Papa A., Lee T.L., Hobbs R.M. (2022). Distinctive molecular features of regenerative stem cells in the damaged male germline. Nat. Commun..

[64] Hussein M.R. (2005). Apoptosis in the ovary: Molecular mechanisms. Hum. Reprod. Update.

[65] Tiwari M., Prasad S., Tripathi A., Pandey A.N., Ali I., Singh A.K., Shrivastav T.G., Chaube S.K. (2015). Apoptosis in mammalian oocytes: A review. Apoptosis.

[66] Iwamoto T., Hiraku Y., Oikawa S., Mizutani H., Kojima M., Kawanishi S. (2004). DNA intrastrand cross-link at the 5′-GA-3′ sequence formed by busulfan and its role in the cytotoxic effect. Cancer Sci..

[67] Guichard N., Bonnabry P., Rudaz S., Fleury-Souverain S. (2017). Stability of busulfan solutions in polypropylene syringes and infusion bags as determined with an original assay. Am. J. Health Syst. Pharm..

[68] Huang Y., Li L. (2013). DNA crosslinking damage and cancer—A tale of friend and foe. Transl. Cancer Res..

[69] Amunugama R., Walter J.C. (2020). A new varietal of DNA interstrand crosslink repair. Cell Res..

[70] Hanawalt P.C. (2002). Subpathways of nucleotide excision repair and their regulation. Oncogene.

[71] Spivak G. (2015). Nucleotide excision repair in humans. DNA Repair.

[72] Duan M., Speer R.M., Ulibarri J., Liu K.J., Mao P. (2021). Transcription-coupled nucleotide excision repair: New insights revealed by genomic approaches. DNA Repair.

[73] Marteijn J.A., Lans H., Vermeulen W., Hoeijmakers J.H. (2014). Understanding nucleotide excision repair and its roles in cancer and ageing. Nat. Rev. Mol. Cell Biol..

[74] Li Q., Yu J.J., Mu C., Yunmbam M.K., Slavsky D., Cross C.L., Bostick-Bruton F., Reed E. (2000). Association between the level of ERCC-1 expression and the repair of cisplatin-induced DNA damage in human ovarian cancer cells. Anticancer. Res..

[75] Gegonne A., Weissman J.D., Zhou M., Brady J.N., Singer D.S. (2006). TAF7: A possible transcription initiation check-point regulator. Proc. Natl. Acad. Sci. USA.

[76] Hayden M.S., Ghosh S. (2014). Regulation of NF-kappaB by TNF family cytokines. Semin. Immunol..

[77] Zhang J., Zhu J., Imrich A., Cushion M., Kinane T.B., Koziel H. (2004). Pneumocystis activates human alveolar macrophage NF-kappaB signaling through mannose receptors. Infect. Immun..

[78] Beattie E.C., Stellwagen D., Morishita W., Bresnahan J.C., Ha B.K., Von Zastrow M., Beattie M.S., Malenka R.C. (2002). Control of synaptic strength by glial TNFalpha. Science.

[79] Mauduit C., Siah A., Foch M., Chapet O., Clippe S., Gerard J.P., Benahmed M. (2001). Differential expression of growth factors in irradiated mouse testes. Int. J. Radiat. Oncol. Biol. Phys..

[80] Hong C.Y., Park J.H., Ahn R.S., Im S.Y., Choi H.S., Soh J., Mellon S.H., Lee K. (2004). Molecular mechanism of suppression of testicular steroidogenesis by proinflammatory cytokine tumor necrosis factor alpha. Mol. Cell Biol..

[81] Jin H., Qiu W.B., Mei Y.F., Wang D.M., Li Y.G., Tan X.R. (2009). Testosterone alleviates tumor necrosis factor-alpha-mediated tissue factor pathway inhibitor downregulation via suppression of nuclear factor-kappa B in endothelial cells. Asian J. Androl..

[82] Antognelli C., Mancuso F., Frosini R., Arato I., Calvitti M., Calafiore R., Talesa V.N., Luca G. (2018). Testosterone and Follicle Stimulating Hormone-Dependent Glyoxalase 1 Up-Regulation Sustains the Viability of Porcine Sertoli Cells through the Control of Hydroimidazolone- and Argpyrimidine-Mediated NF-kappaB Pathway. Am. J. Pathol..

