# Supplementary material for: Busulfan Chemotherapy Downregulates TAF7/TNF-α Signaling in Male Germ Cell Dysfunction
Source: Biomedicines. 2024 Sep 28;12(10):2220. doi: 10.3390/biomedicines12102220 (PMC11504710; doi:10.3390/biomedicines12102220)
Supplement: Supplementary file 1 [file biomedicines-12-02220-s001.zip › biomedicines-3156898-supplementary.pdf]

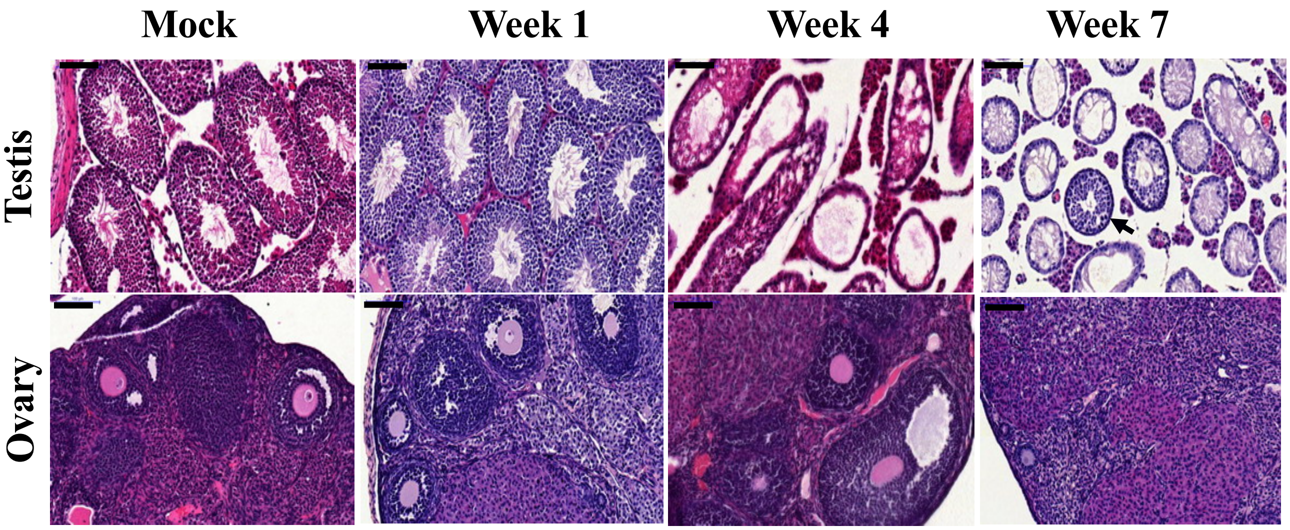

Supplementary Figure S1. Busulfan reduces spermatogenesis and activates apoptosis signaling in the testis.

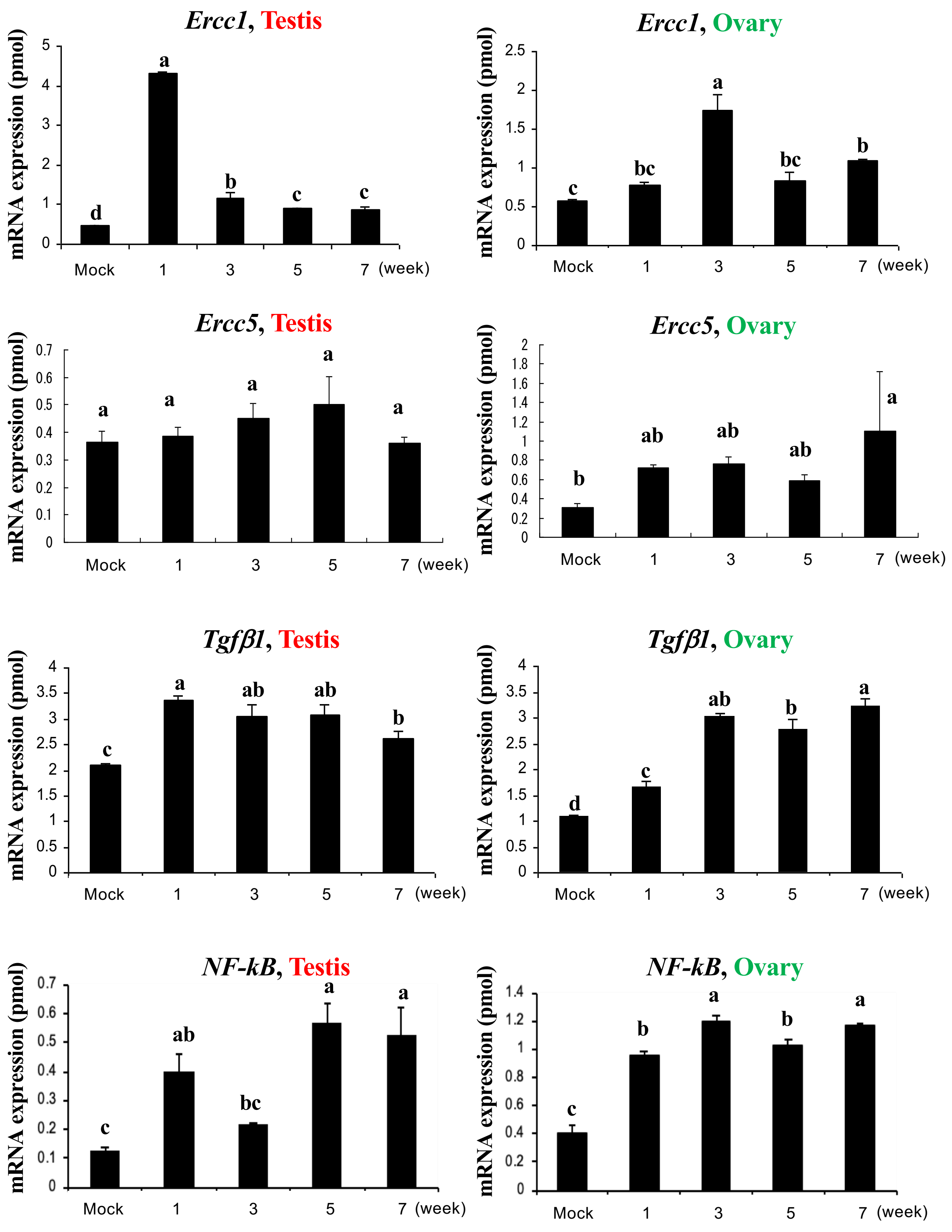

Supplementary Figure S2. Busulfan activates DNA repair signaling in the testis and ovary.

A

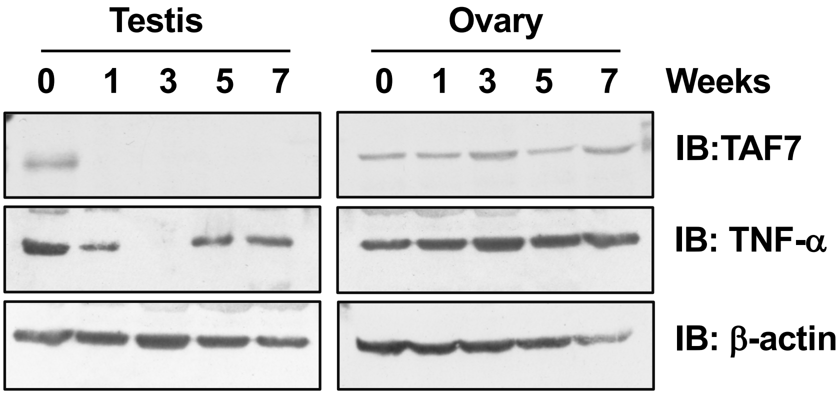

B Mouse *Tnf* gene

```
..........cccgccctcttccccaagggctataaaggcggccgtctgcacagccagcc
AGCAGAAGCTCCCTCAGCGAGGACAGCAAGGGACTAGCCAGGAGGGAGAACAGAAACTCC
AGAACATCTTGGAAATAGCTCCCAGAAAAGCAAGCAGCCAACCAGGCAGGTTCTGTCCCT
TTCACTCACTGGCCCAAGGCGCCACATCTCCCTCCAGAAAAGACACCATG
```

Supplementary Figure S3. Busulfan may downregulate *TAF7* for the reduction in the level of *TNF* expression.

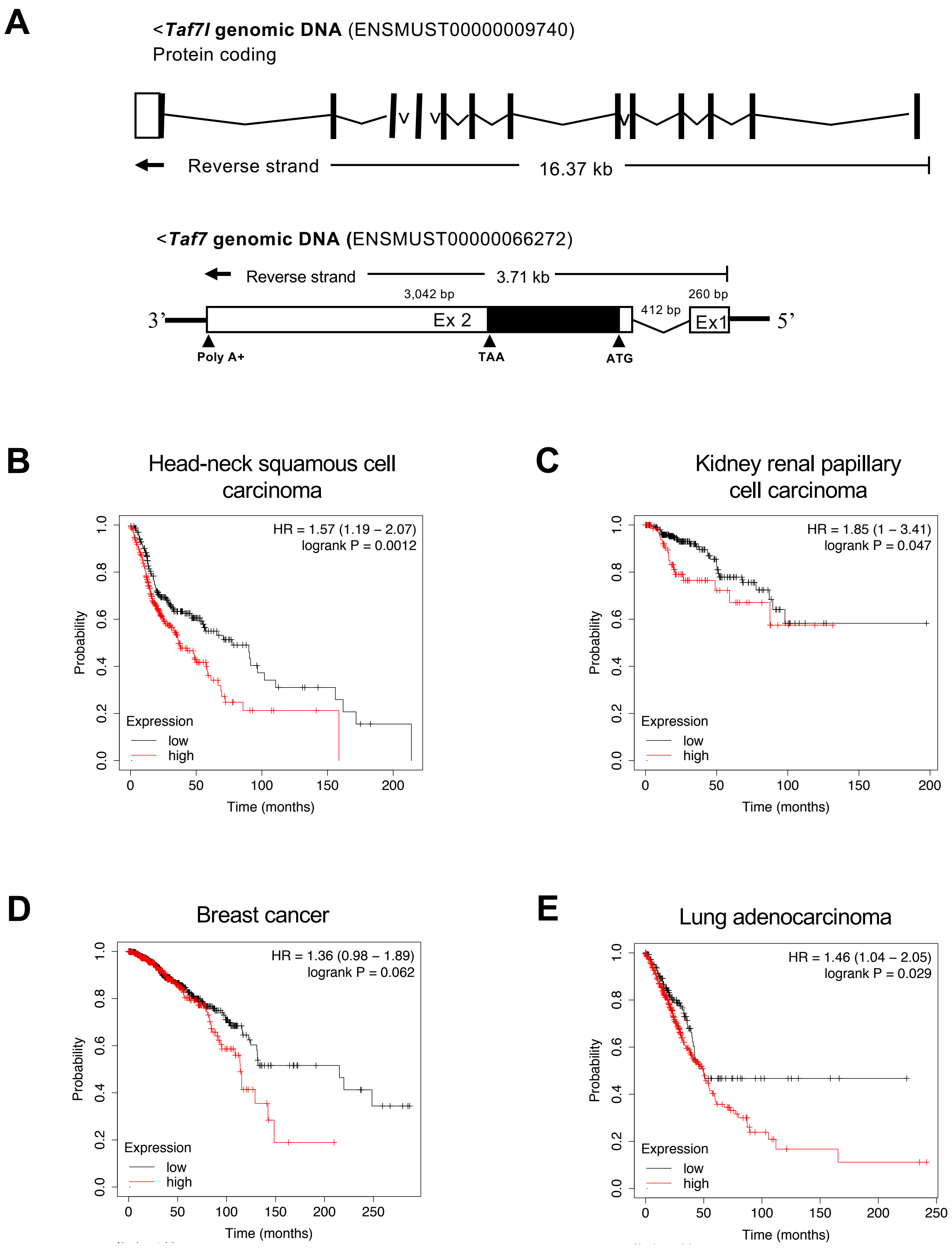

Supplementary Figure S4. *TAF7* is highly expressed in several types of human cancers with poor survival outcomes.

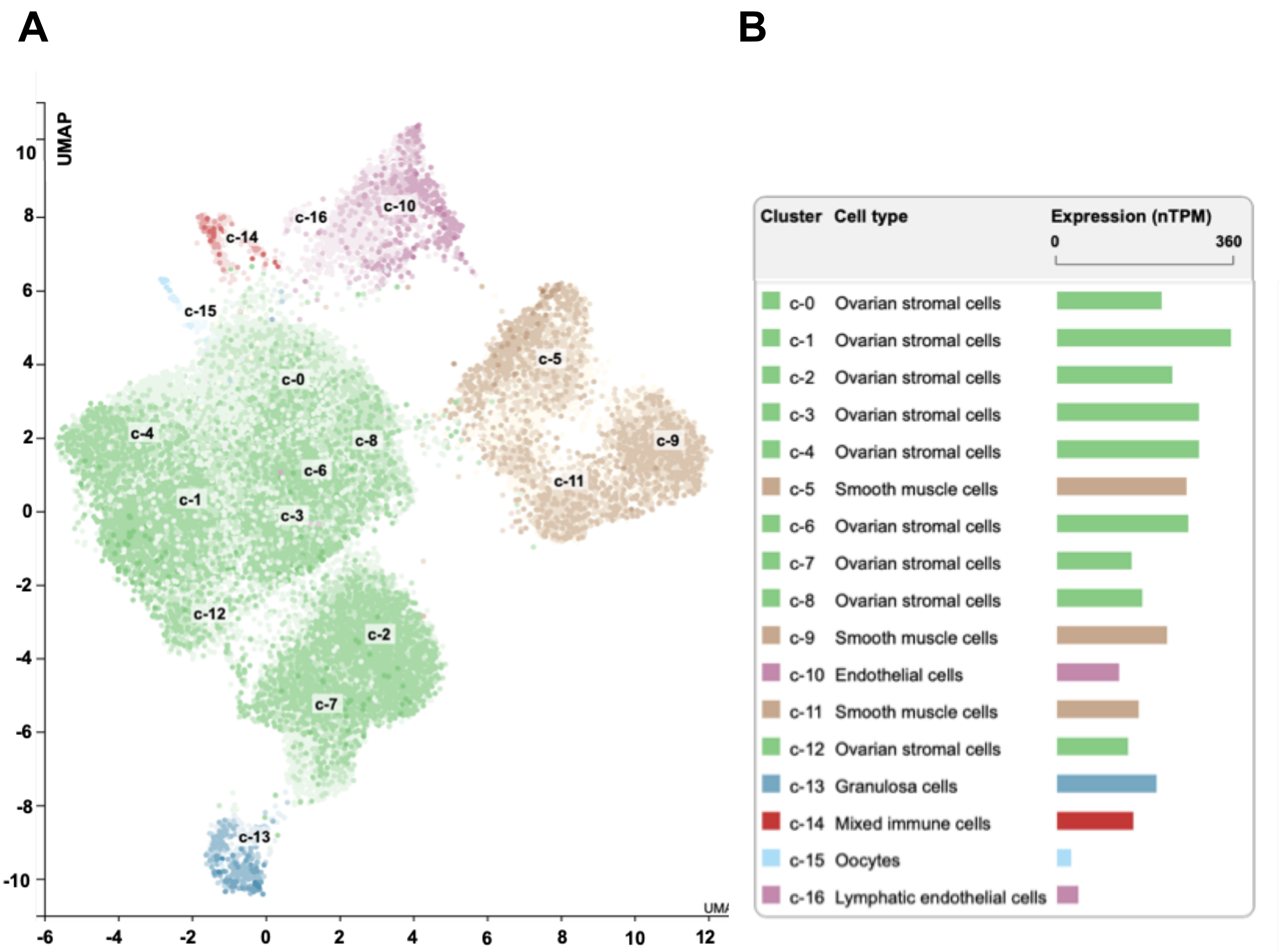

Supplementary Figure S5. *TAF7* is not highly expressed in female germ cells.

Table S1. Primer sequences for qRT-PCR

	Gene	Ref./GenBank	Forward & Reverse	Product	Biomarker	*Reference
1	***TGFβ1***	NM_011577	TGGAGCCTGGACACACAGTA	119	Cell stress	NM_011577
			TAGTAGACGATGGGCAGTGG		Signal transduction	
2	***cKit***	NM_021099	TCCCAGAAACAGGCTGAGTT	121	Spermatogonia	NM_021099
			CATCTTCACGGCAACTGTCA		Oocyte growth	
3	***TP53***	NM_011640	GCCCATGCTACAGAGGAGTC	119	Apoptosis	NM_011640
			GAGTCAGGCCCCACTTTCTT		Cell Cycle Inhibitor	
4	***Msh2***	NM_008628	GGCCAACCAAATACCAACTG	121	Mismatch repair	NM_008628
			CACGTGAATCCCGAAACTCT			
5	***Rb1***	NM_009029	CATGAGAGACCGACATTTGG	121	Cell Cycle	NM_009029
			AGCGTGAGGAAGATCCTTGT		Tumor suppressor	
6	***Tnf***	NM_013693	CAAATGGCCTCCCTCTCAT	121	Growth factor	NM_013693
			AGCTGCTCCTCCACTTGGT		Synaptic plasticity	
7	***Nfkb1***	NM_008689	CACTGCTCAGGTCCACTGTC	120	Intracellular	NM_008689
			CTGAGTTTGCGGAAGGATGT		inflamation	
8	***Cdk4***	NM_009870	GGTACCGAGCTCCTGAAGTTCT	121	Cell Cycling	Custom made
			TCAGAGTTTCCACAGAAGAGAGG			
9	***Ercc1***	NM_007948	CCTGAAAACAGGAGCAAAGTCTA	120	Nuclear Excision	Custom made
			ACATAATCGGGAATCACCTCAC		Repair	
10	***Taf7***	NM_175770	CCGCGAAAAGCTAAGTTCAC	122	TATA-box binding	Custom made
			CTGGGGGCAGTCGTAAGATA		Repair/survival	
11	***Ercc5***	NM_011729	GAAGGCAGAGGAAGGATTCTG	120	Transcription	Custom made
			GCGCTTCATGTCTTGAGCTT		Coupled Repair	

Table S2. Taf7 probe sequence.

Gene	Sieces	Sequense (5' - 3')	mer
TAF7 (NM_175770.4)	Mouse	ACCGCTCCCTGCTGCTTTGCTCAAGCCGCGAAAAGCTAAGTTCACAGT<br>CGCTGGTGAACTGAGCTCACAAGGAATTAAAGATGAGTAAGAACAAA<br>GACGATGCGCCTCATGAGCTAGAGAGC	122

Supplementary Tables S1 and S2
